# Safety profile of chimeric antigen receptor T-cell immunotherapies (CAR-T) in clinical practice

**DOI:** 10.1007/s00228-021-03106-z

**Published:** 2021-02-20

**Authors:** Giulia Bonaldo, Nicola Montanaro, AlbertoVaccheri, Domenico Motola

**Affiliations:** 1 Unit of Pharmacology, Department of Medical and Surgical Sciences, Alma Mater Studiorum University of Bologna, via Irnerio 48, 40126 Bologna, Italy; 2 Pharmacology at the Alma Mater Studiorum, University of Bologna, Bologna, Italy

**Keywords:** CAR-T, Safety profile, Clinical practice, ADR

## Abstract

**Purpose:**

Two chimeric antigen receptor T-cell (CAR-T) therapies have been approved in the United States (USA) in 2017 and Europe (EU) in 2018: axicabtagene ciloleucel and tisagenlecleucel. They contain the patient’s own T cells, which are extracted, genetically modified, and reinfused. Alongside the good efficacy results, the assessment of safety profile of these new therapies represents a great challenge. Our aim was to analyze the reports of the adverse drug reactions (ADR) after CAR-T administration as occurred in the real clinical setting.

**Methods:**

We performed a retrospective observational study, collecting all the reports in EU (EudraVigilance, EV) and US (FAERS) databases of ADRs regarding axicabtagene ciloleucel and tisagenlecleucel. Both descriptive and statistical analyses were performed, the latter by using Reporting Odds Ratio (ROR).

**Results:**

A total number of 1426 reports of suspected ADRs were retrieved in EudraVigilance and FAERS. Patients’ reported age reflected the age range for which the drugs are approved (18–64 years for axicabtagene ciloleucel and patients aged under 25 years for tisagenlecleucel). The most reported event was cytokine release syndrome (CRS), 185 events for tisagenlecleucel and 462 for axicabtagene ciloleucel in FAERS and 137 and 498, respectively, in EudraVigilance. A disproportionality was found comparing axicabtagene ciloleucel with tisagenlecleucel for the above-mentioned event: EV ROR 2.47, 95% CI 2.22–2.74, FAERS 1.89, 1.70–2.10.

**Conclusion:**

CRS represents the major problem with the administration of CAR-T therapies. Our analysis has not revealed new ADRs; however, it supports the safety profile of CAR-T with new data from real clinical setting.

## Introduction

In the last years, personalized medicine in immuno-oncology reached great goals, and, among these, the most important one was the advent of CAR-T therapies. The acronym CAR-T stands for chimeric antigen receptor T-cell therapy, which consists of patient’s genetically modified white blood cells yielded capable to perform antitumor activity using a lentiviral vector encoding an anti-CD19 chimeric antigen receptor-CAR (axicabtagene ciloleucel) or by retroviral transduction to express a chimeric antigen receptor (tisagenlecleucel). After lymphodepletion, the modified T cells reinfused in the patients can attach a specific antigen on the tumor cells. Two CAR-T therapies have been approved in Europe (August 2018), i.e., axicabtagene ciloleucel (Yescarta®) [1, 2] and tisagenlecleucel (Kymriah®) [3]. These therapies have been also approved in 2017 by the US Food and Drug Administration (US FDA) [4, 5].

Axicabtagene ciloleucel is approved for two types of blood cancer: diffuse large B-cell lymphoma (DLBCL) and primary mediastinal large B-cell lymphoma (PMBCL) [6]. Tisagenlecleucel is approved for the treatment of B-cell acute lymphoblastic leukemia (ALL) refractory or in relapse (post-transplant, second or later relapse) in children and young adults up to 25 years and for adult patients with relapsed or refractory DLBCL after two or more lines of systemic therapy [7]. Two main studies were performed with tisagenlecleucel in B-cell ALL (92 children and young adults) [8] and in DLBCL (165 patients) [9]. Twelve months after treatment, the survival probability was 70% and 40.2%, respectively [10]. A main study in 111 patients with DLBCL and PMBCL was carried out for axicabtagene ciloleucel [11], showing a complete response in 47% of the patients and a partial response in the 19% [12]. Apart from the great efficacy results, the knowledge of the safety profile of these new therapies represents a great challenge. Both drugs must be administered in a qualified center for hematological malignancies with healthcare professionals trained in the management of possible adverse events. Patients undergo pre-medication, clinical assessment prior to infusion, and monitoring after infusion to minimize potential adverse events. Our aim was to evaluate all the adverse events reported following the administration of axicabtagene ciloleucel and tisagenlecleucel in the real clinical setting in Europe and in the USA.

## Methods

Data were retrieved from the European database of suspected adverse drug reaction reports (EudraVigilance) using the online interface adrreports.eu [13] and from the US Food and Drug Administration Adverse Event Reporting system (FAERS) using a freely available and of public consultation dashboard. EudraVigilance is the system for the management and collection of suspected adverse events reported for medicines. It is maintained by the European Medicines Agency (EMA) on behalf of the European Union (EU). In November 2017, the new EudraVigilance system with enhanced functionalities was launched with an improvement of the collection of the individual case safety reports (ICSRs) from regulatory authorities and marketing authorization holders (MAHs) and of the detection of possible safety signals [14]. The FDA Adverse Event Reporting System (FAERS) is the database for the collection of all the reports of suspected ADRs submitted to FDA. All the reports are in a structured form that includes administrative information, patient’s characteristics, suspected and concomitant drugs, and the adverse drug reactions. For our aim, we focused on the analysis of both anti-CD19 chimeric antigen receptor T-cell immunotherapies (CAR-T). We retrieved all the reports with axicabtagene ciloleucel or tisagenlecleucel reported as suspected drugs. Considering that both drugs received a marketing authorization valid throughout the European Union on 23 August 2018, we considered the period between September 2018 and 22 October 2019 for our analysis in EudraVigilance. For FAERS analysis, we considered the period between September 2017 (first CAR-T approval date) and 30 June 2019 (date of the most recent update of the dashboard).

### Descriptive analysis

The extracted reports were identified by a unique EU Local Number in EudraVigilance or by a unique Case ID in FAERS. The information reported was the report type (spontaneous or from clinical studies), receipt date, primary source qualification (healthcare professional or non-healthcare professional), patient age group, patient sex, preferred term MedDRA, seriousness criteria, and suspect and concomitant drugs. The MedDRA is a standardized medical terminology that allows to report adverse events in the same way all over the world. It is used by both databases, and this allows an easy comparison of the data reported [15]. This dictionary has a hierarchic terminology: several preferred terms (PTs) are grouped in one System Organ Class (SOC) by etiology, manifestation site, or purpose.

We analyzed all the reports related to axicabtagene ciloleucel or tisagenlecleucel. A descriptive analysis was performed to evaluate all the reported adverse events. It should be noted that each report could contain one or more adverse events. For each drug, it was checked if the reported events were listed in the corresponding Summary of the Product Characteristics (SPCs) to ascertain the notoriety of the adverse reactions.

### Statistical analysis

A case-non-case analysis was performed by using the Reporting Odds Ratio (ROR) with 95% confidence interval as statistical parameter. ROR allows a quantitative approach by the use of contingency tables. The aim is to compare the frequency of a drug-reaction pair with all the others in the database or with different therapeutic regimens. If ROR is >1, an increased frequency for the drug-reaction pair can be assumed. Considering the specificity of the treatments analyzed, the use of the whole database was considered not appropriate. Therefore, a disproportionality analysis was carried out by comparing the two treatments. Other articles in literature have used a similar approach when comparing different therapeutic regimens [16]. The proportions of the adverse drug reactions to axicabtagene ciloleucel were compared to those with tisagenlecleucel. The denominator was the total number of adverse drug reactions reported for both drugs.

## Results

Table 1 shows the most reported adverse events for both CAR-T therapies in EudraVigilance and FAERS. For each event, it is also indicated whether the ADR is reported in the Summary of Product Characteristic of the corresponding CAR-T therapy (pointed out with *); it is not directly reported in the SPC but linked to other reported events (#), or it is not reported at all.

**Table 1:** Most reported adverse events for CAR-T therapies in EudraVigilance and FAERS

Tisagenlecleucel						Axicabtagene ciloleucel					
FAERS			EudraVigilance			FAERS			EudraVigilance		
Events	*N*	%	Events	*N*	%	Events	*N*	%	Events	*N*	%
*Cytokine release syndrome	185	8.16	*Cytokine release syndrome	137	8.58	*Cytokine release syndrome	462	14.37	*Cytokine release syndrome	498	18.79
*Pyrexia	124	5.47	*Pyrexia	90	5.64	*Neurotoxicity	312	9·70	*Neurotoxicity	247	9·32
Malignant neoplasm progression	61	2.69	*Hypotension	49	3.07	*Pyrexia	204	6.35	*Pyrexia	187	7.06
*Neurotoxicity	60	2.65	*Neurotoxicity	43	2.69	*Encephalopathy	110	3.42	*Encephalopathy	94	3.55
*Hypotension	53	2.34	Malignant neoplasm progression	37	2.32	*Hypotension	89	2.77	*Hypotension	75	2.83
Acute lymphocytic leukemia recurrent	40	1.76	Acute lymphocytic leukemia recurrent	33	2.07	*Aphasia	66	2.05	*Tachycardia	63	2.38
*Headache	32	1.41	*White blood cell count decreased	24	1.5	*Tachycardia	63	1.96	#CAR T-cell encephalopathy syndrome	53	2.0
*Tachycardia	31	1.37	*Febrile neutropenia	22	1.38	**#**Confusional state	58	1.80	*Aphasia	44	1.66
*Febrile neutropenia	29	1.28	*Hypoxia	21	1.31	*Neutropenia	57	1.77	**#**Confusional state	43	1.62
*Neutrophil count decreased	27	1.19	*Platelet count decreased	20	1.25	*Tremor	54	1.68	*Neutropenia	43	1.62
*Hypoxia	27	1.19	*Headache	19	1.19	*Headache	41	1.28	*Tremor	41	1.55
*Fatigue	25	1.10	*Neutrophil count decreased	19	1.19	#Somnolence	41	1.28	*Fatigue	38	1.43
Drug ineffective	23	1.01	Diffuse large B-cell lymphoma	18	1.13	#Car T-cell encephalopathy syndrome	41	1.28	*Headache	38	1.43
*Coagulopathy	23	1.01	*Acute kidney injury	17	1.06	*Hypoxia	39	1.21	*Hypoxia	33	1.25
*Platelet count decreased	22	0.97	*Tachycardia	17	1.06	*Fatigue	37	1.15	*Thrombocytopenia	30	1.13
*Confusional state	18	0.79	Acute lymphocytic leukemia	16	1.0	*Thrombocytopenia	36	1.12	**#**Somnolence	28	1.06
*Neutropenia	17	0.75	*Coagulopathy	15	0.94	*Disorientation	28	0.87	Diffuse large B-cell lymphoma	25	0.94
*Hypogammaglobulinaemia	17	0.75	*Encephalopathy	15	0.94	*Atrial fibrillation	26	0.81	Death	24	0.91
*Encephalopathy	16	0.71	*Blood fibrinogen decreased	14	0.88	**#**Mental status changes	26	0.81	*Malaise	24	0.91
#C-Reactive protein increased	16	0.71	Drug ineffective	14	0.88	Disease progression	26	0.81	Disease progression	19	0.72
*Serum ferritin increased	16	0.71	*Fatigue	14	0.88	*Nausea	25	0.78	*Disorientation	18	0.68
Death	15	0.66	*Lymphopenia	14	0.88	#Pancytopenia	23	0.72	**#**Mental status changes	18	0.68
*Nausea	15	0.66	*Serum ferritin increased	14	0.88	*Febrile neutropenia	23	0.72	*Atrial fibrillation	16	0.6
Diffuse large B-cell lymphoma	14	0.62	#C-reactive protein increased	13	0.81	*Malaise	22	0.68	*Chills	16	0.6
*Cytopenia	14	0.62	Sepsis	13	0.81	*Vomiting	20	0.62	*Nausea	16	0.6
Acute lymphocytic leukemia	14	0.62	Disease progression	12	0.75	*Agitation	20	0.62	*Agitation	15	0.57
*Dyspnea	13	0.57	*Nausea	12	0.75	Diffuse large B-cell lymphoma	20	0.62	**#**Pancytopenia	15	0.57
*Respiratory failure	13	0.57	*Confusional state	11	0.69	Death	18	0.56	*Hypogammaglobulinaemia	14	0.53
*Diarrhea	12	0.53	*Neutropenia	11	0.69	#Memory impairment	17	0.53	Dysgraphia	13	0.49
*Acute kidney injury	12	0.53	*Disseminated intravascular coagulation	10	0.63	*Chills	17	0.53	*Vomiting	13	0.49
#White blood cell count decreased	12	0.53	*Anemia	9	0.56	Dysphagia	17	0.53	C-reactive protein increased	12	0.45
Therapy non-responder	12	0.53	#B-cell aplasia	9	0.56	**#**Transaminases increased	17	0.53	*Diarrhea	12	0.45
#Malaise	11	0.49	*Pancytopenia	9	0.56	*Hemophagocytic lymphohistiocytosis	17	0.53	Dysphagia	12	0.45
B-cell type acute leukemia	11	0.49	Death	8	0.5	Dysgraphia	17	0.53	**#**Memory impairment	12	0.45
*Cough	10	0.44	*Lymphocyte count decreased	8	0.5	*Pleural effusion	16	0.50	**#**Neutrophil count decreased	12	0.45
*Vomiting	10	0.44	*Multiple organ dysfunction syndrome	8	0.5	*Hyponatremia	14	0.44	*Seizure	12	0.45
*Multiple organ dysfunction syndrome	10	0.44	*Respiratory failure	8	0.5	Incontinence	14	0.44	**#**Tachypnea	12	0.45
*Fluid overload	10	0.44	*Chills	7	0.44	C-Reactive protein increased	14	0.44	**#**Transaminases increased	12	0.45
*Pain	9	0.40	*Hypertension	7	0.44	*Anemia	13	0.40	*Anemia	11	0.42
*Mental status changes	9	0.40	*International normalized ratio increased	7	0.44	*Seizure	13	0.40	**#**Bone marrow failure	11	0.42
*Decreased appetite	9	0.40	Pneumonia	7	0.44	*Abdominal pain	13	0.40	*Infection	11	0.42
*Seizure	9	0.40	*Abdominal pain	6	0.38	**#**Tachypnea	13	0.40	**#**White blood cell count decreased	11	0.42
*Pancytopenia	9	0.40	#Prolonged partial thromboplastin time	6	0.38	*Cytopenia	13	0.40	Incontinence	10	0.38
*Tremor	9	0.40	*Blood creatinine increased	6	0.38	*Cardiac arrest	11	0.34	Muscular weakness	10	0.38
*Chills	9	0.40	*Diarrhea	6	0.38	#Asthenia	11	0.34	*Pleural effusion	10	0.38
Disease progression	9	0.40	*Fibrin D dimer increased	6	0.38	*Infection	11	0.34	*Cytopenia	9	0.34
*Delirium	9	0.40	*Fluid overload	6	0.38	Sepsis	10	0.31	*Febrile neutropenia	9	0.34
Sepsis	8	0.35	*Hemoglobin decreased	6	0.38	Pneumonia	10	0.31	*Acute kidney injury	8	0.3
*Myalgia	8	0.35	*Malaise	6	0.38	*Delirium	10	0.31	**#**Depressed level of consciousness	8	0.3
*Somnolence	8	0.35	*Mental status changes	6	0.38	#White blood cell count decreased	10	0.31	Disseminated intravascular coagulation	8	0.3

*N* number of reports

*Event listed in the Summary of Product Characteristic (SPC)

#Event not reported in the SPC as such but linked to other listed events

### Descriptive analysis of axicabtagene ciloleucel

#### EudraVigilance

We retrieved 683 reports of suspected adverse reactions referred to axicabtagene ciloleucel. One hundred ninety-five reports were related to female patients (28.6%) and 306 (44.8%) to males. For 182 reports (26.6%), sex of the patients was not reported. Table 2 shows the reports classified by age and sex in the two databases. Most of the patients (277; 40.6%) belonged to the 18–64 years age class. Only one report concerned a female patient of the age class 12–17 years and 2 patients, one male and one female, aged >85 years. The great majority of the ADRs (673, 98.5%) were reported by healthcare professionals. Overall, 2650 adverse events were reported for axicabtagene ciloleucel. The top five most reported were cytokine release syndrome (498 events, 18.8%), neurotoxicity (247, 9.3%), pyrexia (187, 7.1%), encephalopathy (94, 3.6%), and hypotension (75, 2.8%). The most reported concomitant therapies were acyclovir, levetiracetam, and ondansetron and, to a less extent, tocilizumab.

**Table 2:** Patients’ age and sex of adverse events occurring in CAR-T therapies performed in real clinical setting

**EUDRAVIGILANCE**							**FAERS**						
	**Age**	**Sex**	**Subtotal**	**%**	**Total**	**%**		**Age**	**Sex**	**Subtotal**	**%**	**Total**	**%**
Tisagenlecleucel	2 months - 2 years	F	5	1.76	11	3.87	Tisagenlecleucel	2 months - 2 years	F	4	1.01	12	3.02
		M	5	1.76					M	5	1.26		
		NA	1	0.35					NA	3	0.75		
	3-11 Years	F	19	6.69	53	18.66		3-11 years	F	26	6.53	78	19.60
		M	31	10.92					M	48	12.06		
		NA	3	1.06					NA	4	1.01		
	12-17 Years	F	23	8.10	55	19.37		12-17 Years	F	25	6.28	60	15.08
		M	28	9.86					M	30	7.54		
		NA	4	1.41					NA	5	1.26		
	18-64 Years	F	39	13.73	93	32.75		18-64 Years	F	36	9.05	91	22.86
		M	47	16.55					M	51	12.81		
		NA	7	2.46					NA	4	1.01		
	65-85 Years	F	4	1.41	19	6.69		65-85 Years	F	4	1.01	24	6.03
		M	14	4.93					M	20	5.03		
		NA	1	0.35					NA	0	0.00		
	> 85 Years	F	0	0.00	0	0.00		> 85 Years	F	0	0	0	0.00
		M	0	0.00					M	0	0		
		NA	0	0.00					NA	0	0		
	Not Specified	F	13	4.58	53	18.66		Not Specified	F	26	6.53	133	33.42
		M	22	7.75					M	45	11.31		
		NA	18	6.34					NA	62	15.58		
		**Total**	**284**	**100**	**284**	**100**			**Total**	**398**	**100**	**398**	**100**
	**Age**	**Sex**	**Subtotal**	**%**	**Total**	**%**		**Age**	**Sex**	**Subtotal**	**%**	**Total**	**%**
**Axicabtagene ciloleucel**	2 months - 2 years	F	0	0	0	0.00	**Axicabtagene ciloleucel**	2 months - 2 years	F	0	0	0	0.00
		M	0	0					M	0	0		
		NA	0	0					NA	0	0		
	3-11 years	F	0	0	0	0.00		3-11 years	F	0	0	0	0.00
		M	0	0					M	0	0		
		NA	0	0					NA	0	0		
	12-17 Years	F	1	0.15	1	0.15		12-17 Years	F	0	0	0	0.00
		M	0	0					M	0	0		
		NA	0	0					NA	0	0		
	18-64 Years	F	92	13.47	277	40.56		18-64 Years	F	118	15.88	337	45.36
		M	165	24.16					M	208	27.99		
		NA	20	2.93					NA	11	1.48		
	65-85 Years	F	62	9.08	149	21.81		65-85 Years	F	66	8.88	202	27.19
		M	81	11.86					M	125	16.82		
		NA	6	0.87					NA	11	1.48		
	> 85 Years	F	1	0.15	2	0.30		> 85 Years	F	2	0.27	2	0.27
		M	1	0.15					M	0	0		
		NA	0	0					NA	0	0		
	Not Specified	F	39	5.71	254	37.19		Not Specified	F	40	5.38	202	27.19
		M	59	8.64					M	54	7.27		
		NA	156	22.84					NA	108	14.54		
		**Total**	**683**	**100**	**683**	**100**			**Total**	**743**	**100**	**743**	**100.00**

#### FAERS

We retrieved 743 reports of suspected adverse reactions referred to axicabtagene ciloleucel in FAERS. Overall, one report (0.1%) was reported in 2017, 440 (59.2%) in 2018, and 302 (40.7%) in 2019 until the 30th of June. Report for male patients were 387 (52.1%) and those for females were 226 (30.4%). Sex was not stated in 130 reports (17.5%). The great majority of the reports were related to 18–64 years age class (45.4%). Only two reports concerned female patients aged >85 years. Table 2 shows reports classified by age and sex in the two databases. The vast majority of reporters were healthcare professionals (699, 94.08%), whereas 18 reports (2.42%) came from consumers. Overall, 3215 events were reported for axicabtagene ciloleucel in FAERS. The top five most reported events were cytokine release syndrome (462 events, 62.2%), neurotoxicity (312, 42.0%), pyrexia (204, 27.5%), encephalopathy (110, 14.8%), and hypotension (89, 11.98%). The most reported concomitant therapies were acyclovir, levetiracetam, allopurinol, fluconazole, fludarabine, cefepime, vancomycin, cyclophosphamide, ondansetron, and tocilizumab.

### Descriptive analysis of tisagenlecleucel

#### EudraVigilance

In the same period, we retrieved 284 reports of suspected adverse reactions referred to tisagenlecleucel of which 137 in 2018 and 147 in 2019. One hundred and three (36.3%) were related to female patients and 147 (51.8%) to males. For 34 reports (12.0%), sex of the patients was not stated. All data are shown in Table 2. Overall, 119 reports regarded patients aged less than 18 years. In particular, 11 patients (3.9%) were aged 2 months–2 years, 54 (18.7%) 3–11 years, and 55 (19.4%) 12–17 years. Ninety-three reports (32.7%) referred to the age class 18–64 years. The large majority (96.1%) of the reports came from healthcare professionals. Overall, 1597 events were reported for tisagenlecleucel. The top five most reported events were cytokine release syndrome (137 events, 8.6%), pyrexia (90, 5.6%), hypotension (49, 3.1%), neurotoxicity (43, 2.7%), and malignant neoplasm progression (37, 2.3%). The most reported concomitant therapies were cyclophosphamide, fludarabine, levetiracetam, acyclovir, amlodipine, ondansetron, and tocilizumab.

#### FAERS

Overall, 398 reports were retrieved for tisagenlecleucel in FAERS. Of these, 10 (2.5%) were reported in 2017, 179 (45.0%) in 2018, and 209 (52.5%) in 2019 until June 30. The half of the reports (199, 50.0%) concerned male patients, 121 (30.4%) females, and for 78 (19.6%), sex was not reported. The majority of the reports were related to minors, as shown in Table 2. Of the 91 patients in the age class 18–64 years, 54 (59.3%) were patients ≤ 25 years old. For 133 (33.4%) patients, age class was not specified. The majority of reports (79.4%) came from healthcare professionals, 20.4% from consumers. The overall events reported for tisagenlecleucel in FAERS were 2268. The top five most reported ones were cytokine release syndrome (185, 46.5%), pyrexia (124, 31.2%), malignant neoplasm progression (61, 15.3%), neurotoxicity (60, 15.1%), and hypotension (53, 13.3%). The most reported concomitant medications were levetiracetam, acyclovir, allopurinol, fludarabine, voriconazole, cyclophosphamide, cefepime, cotrimoxazol, ondansetron, levofloxacin, and tocilizumab.

### Statistical analysis

We compared the ADRs reported for axicabtagene ciloleucel with those for tisagenlecleucel in either database. Overall, 196 events were reported for both drugs in EudraVigilance and 243 in FAERS. A disproportionality was found for axicabtagene ciloleucel as compared to tisagenlecleucel concerning the following three events. The ROR values were cytokine release syndrome (EV ROR 2.47 [95% CI 2.22–2.74], FAERS ROR 1.89 [1.70–2.10]), neurotoxicity (EV ROR, 3.71 [3.13–4.41], FAERS ROR 3.96 [3.42–4.57]), and pyrexia (EV ROR 1.27 [1.08–1.50], FAERS ROR 1.17 [1.00–1.37]). All these three events are acknowledged on the Summary of Product Characteristics (SPC) of the drugs. As shown in Table 3, among the drug-reaction pairs with higher and statistically significant ROR in both databases, we found aphasia (EV ROR 8.97 [3.47–23.22], FAERS ROR 15.82 [6.46–38.77]), CAR-T-cell-related encephalopathy syndrome (EV ROR 8.13[3.80–17.40], FAERS ROR 9.75 [3.73–25.49]), atrial fibrillation (EV ROR 4.84 [1.11–21.11], FAERS ROR 9.24, [95% CI 2.36–36.20]), and thrombocytopenia (EV ROR 4.56 [95% CI 1.95–10.67], FAERS ROR 5.13 [95% CI 2.49–10.53]).

**Table 3:** Adverse events reported with axicabtagene compared to tisagenlecleucel in EudraVigilance and FAERS and Reporting Odds Ratio (ROR)

EUDRAVIGILANCE					
Events	N axicabtagene	N tisagenleucleucel	ROR	CI_low	CI_up
Aphasia	44	3	8.97	3.47	23.22
Tremor	41	3	8.35	3.19	21.84
CART-cell-related encephalopathy syndrome	53	4	8.13	3.80	17.40
Atrialfibrillation	16	2	4.84	1.11	21.11
Thrombocytopenia	30	4	4.56	1.95	10.67
Encephalopathy	94	15	3.88	2.78	5.42
Neurotoxicity	247	43	3.71	3.13	4.41
Somnolence	28	5	3.40	1.58	7.30
Disorientation	18	4	2.72	1.05	7.07
Cytokinereleasesyndrome	498	137	2.47	2.22	2.74
Malaise	24	6	2.42	1.17	5.02
Confusionalstate	43	11	2.38	1.47	3.84
Neutropenia	43	11	2.38	1.47	3.84
Tachycardia	63	17	2.26	1.57	3.26
Fatigue	38	14	1.64	1.04	2.61
Pyrexia	187	90	1.27	1.08	1.50
FAERS					
Events	N axicabtagene	N tisagenleucleucel	ROR	CI_low	CI_up
Aphasia	66	3	15.82	6.46	38.77
Car T-Cell-Related Encephalopathy Syndrome	41	3	9.75	3.73	25.49
Atrial Fibrillation	26	2	9.24	2.36	36.20
Thrombocytopenia	36	5	5.13	2.49	10.53
Encephalopathy	110	16	4.99	3.65	6.80
Disorientation	28	4	4.97	2.10	11.77
Hyponatraemia	14	2	4.96	1.10	22.33
Incontinence	14	2	4.96	1.10	22.33
Tremor	54	9	4.29	2.64	6.97
Transaminases Increased	17	3	4.01	1.30	12.43
Neurotoxicity	312	60	3.96	3.42	4.57
Somnolence	41	8	3.65	2.10	6.34
Neutropenia	57	17	2.39	1.64	3.48
Agitation	20	6	2.36	1.10	5.08
Confusional State	58	18	2.30	1.59	3.32
Mental Status Changes	26	9	2.05	1.12	3.74
Disease Progression	26	9	2.05	1.12	3.74
Cytokine Release Syndrome	462	185	1.89	1.70	2.10
Tachycardia	63	31	1.44	1.06	1.97
Pyrexia	204	124	1.17	1.00	1.37

*N* number of reports, *ROR* reporting odds ratio, *CI_low* lower bound of the 95% confidence interval, *CI_up* upper bound of the 95% confidence interval

## Discussion

To the best of our knowledge, this is the first study aimed at evaluating and comparing the safety profile of the CAR-T therapies with the use of data of real clinical practice. Post-approval pharmacovigilance is considered crucial for the evaluation of CAR-T safety profile [17], as it allows long-term follow-up in a large and uncontrolled population. From the present analysis, no new and unexpected ADRs emerged in daily clinical practice, and this is reassuring. The patients experiencing an ADR with either drug were different in term of age. Most of axicabtagene ciloleucel patients were aged 18–64 years or more, whereas those receiving tisagenlecleucel were under 25 years of age and mainly minors. This reflects the indications for which the drugs have been approved: tisagenlecleucel is the only CAR-T therapy specifically approved for the treatment of patients ≤25 years of age with R/R B-cell acute lymphoblastic leukemia.

Proportion of male patients was higher with both drugs and databases. This does not point out a greater predisposition of male patients to develop an ADR, but it is rather related to the prevalence of the disease to be treated. Both diffuse large B-cell lymphoma (DLBCL) and acute lymphocytic leukemia (ALL) have a slightly higher incidence in males than females. The US National Cancer Institute reported that the number of new cases per 100,000 persons in the period 2012–2016 were 6.7 in males and 4.6 in female for DLBCL and 1.9 and 1.5, respectively, in female for ALL [18, 19].

A lower presence of consumers as reporters has been identified in EudraVigilance in comparison with FAERS, this confirming the lower tendency of patients to report ADRs in Europe [20]. Cytokine release syndrome (CRS) was the most reported ADR in both databases and for both drugs.

The pathophysiology of the syndrome is unclear, and recent studies showed that IL-6, IL-1, and nitric oxide produced by macrophages are involved in its course [21]. The vast majority of the others events reported in our study could be symptoms of this syndrome, which is characterized mainly by pyrexia and hypotension, the most reported events retrieved after CRS. Other ADRs associable with CRS are hypoxia, chills, cardiac adverse events (e.g., tachycardia, atrial fibrillation, and cardiac arrest), acute kidney injury, and also hepatic and musculoskeletal toxicities [22]. All these events have been reported for both drugs and in both databases. Different studies in literature account these events as the most common with CAR-T therapies [23–25]. In most cases, the trigger is the cytokine release syndrome that starts a subsequent cascade of events. The main goal is to prevent or at least limit the CRS to a low grade [26, 27]. Some strategies in the management of CAR-T-related toxicities have been described such as pharmacological immunosuppression with tocilizumab or corticosteroids, suicide or elimination genes, and targeted activation [28, 29]. Recently, Lee at al. have described new methods that could prevent or rather terminate within 3 h the CRS-like toxicity by using low molecular weight adapters [30].

To date, IL-6 receptor antagonist tocilizumab represents the elective therapy in the management of the CRS. It was reported as concomitant drug in several reports of our survey, but it should be better considered as an emergency measure. As reported in its SPC, a minimum of four doses of tocilizumab should be available prior to infusion and during the monitoring period for use in the event of CRS. Neurotoxicity was the second most reported ADR, also referred to as CART-related encephalopathy syndrome. Several events reported in our analysis are symptoms of this toxicity such as encephalopathy, seizures, headache, aphasia, and memory impairment [31, 32]. A disproportionality has been highlighted for axicabtagene ciloleucel compared to tisagenlecleucel for both the above-mentioned events: cytokine release syndrome and neurotoxicity. These data may suggest a higher association of these events to axicabtagene ciloleucel as compared to tisagenlecleucel. However, it should be remembered that the target population is different for the two CAR-T therapies, and this could influence the pattern and frequencies of the ADRs. Some rare and serious events have been reported such as multiple organ dysfunction syndrome, coagulopathies, included disseminated intravascular coagulation, and hemophagocytic lymphohistiocytosis. The complexity of the main event reported after the CAR-T treatment, i.e., the CRS, could also be at the root of these life-threatening ADRs [22, 33]. Inflammatory cells during CRS may lead to destruction of the integrity of endothelial barrier, thus causing CRS-related coagulopathy [34].

Among the most reported events, we found other hematological disorders: neutrophil count decreased, platelet count decreased, neutropenia, cytopenia, white blood cell count decreased, pancytopenia, and others. More than true adverse reactions to treatment, these are events related to the administration schedule, such as lymphodepleting chemotherapy with cyclophosphamide and fludarabine and leukapheresis before CAR-T infusions are the main culprits of these adverse reactions. As for the drugs reported as concomitant, some of them were drugs used in the administration procedure. For example, paracetamol and diphenhydramine are often used for pre-medication, allopurinol are used to reduce uric acid level in order to prevent tumor lysis syndrome in patient at risk, and corticosteroids and anti-seizure medicines such as levetiracetam are used for seizure prophylaxis. Some events related to the potential inefficacy of the treatment were also found, such as malignant neoplasm progression, acute lymphocytic leukemia recurrence, drug ineffectiveness (mainly for tisagenlecleucel), diffuse large B-cell lymphoma, acute lymphocytic leukemia, and B-cell-type acute leukemia. All these suggest the importance of continuing to evaluate data on approved drugs during their use in clinical practice. Our study also showed that the frequencies of the reported events were about the same for the two drugs in both databases. However, as shown in Table 1, they were lower than the frequencies highlighted in pre-marketing clinical trials. According to the safety information about axicabtagene ciloleucel—Yescarta®—in the ZUMA-1 study (108 patients enrolled), the most frequent ADRs were cytokine release syndrome (93%), encephalopathy (58%), and infections (39%). Other ADRs reported as very common (≥ 1/10) in the SPC are hematological disorders (leukopenia, neutropenia, anemia, and thrombocytopenia), tachycardia, arrhythmia, headache, tremor, and dizziness [6]. The safety assessment of tisagenlecleucel—Kymriah®—was based on a total of 194 patients belonging to two clinical multicentre studies: CCTL019B2202 and CCTL019C2201. The most common ADRs were cytokine release syndrome (77% in CCTL019B2202, 57% in CCTL019C2201) and infections (73% in CCTL019B2202, 58% in CCTL019C2201); instead frequent hematological adverse reactions were decrease in lymphocytes (100% in both the studies) and decrease in neutrophils (100% in CCTL019B2202, 97% in CCTL019C2201) [7].

These differences are probably due to the well-known limitation of ADR under-reporting in clinical practice [35, 36] that represents one of the limitations of our study. Furthermore, the same ADRs could have been reported with different terminology, and this may have led to distortion of their frequencies. In addition, the reports of suspected ADRs may be often incomplete and of poor quality, making difficult to obtain satisfactory data. This also made difficult the detection of duplicates. Furthermore, this type of study does not allow the quantification of the risk of an adverse event (in the absence of information about the number of exposed patients) but only gives a frequency description and suggests a possible association between drugs and adverse events. In any case, pharmacovigilance tools are used worldwide also by regulatory agencies to perform signal detection (i.e., the screening of safety information related to new ADR or to changed frequencies for already known ones) and are undoubtedly valuable, since they provide the highest volume of information at the lowest cost [37].

## Conclusion

The type of adverse reactions reported from clinical practice is consistent with those in the SPCs of either drugs. The analysis of two of the largest databases worldwide such as EudraVigilance and FAERS has not revealed new signal, i.e., no new potential ADRs or increased frequencies. With the increasing use of these new therapies, a better definition of their real safety profile will be possible.

## Data Availability

For this type of study, formal consent is not required.
